# Emerging needs of social innovators and social innovation ecosystems

**DOI:** 10.1007/s11365-021-00789-9

**Published:** 2021-11-13

**Authors:** David B. Audretsch, Georg M. Eichler, Erich J. Schwarz

**Affiliations:** 1 Department of Innovation Management and Entrepreneurship, Alpen-Adria Universität Klagenfurt, 9020 Klagenfurt, Austria; 2 School of Public and Environmental Affairs, Indiana University Bloomington, 1315 E. 10th Avenue SPEA, Bloomington, IN 47405 USA

**Keywords:** Social innovation, Needs of social innovators, Social entrepreneurship, Ecosystems

## Abstract

Social innovations (SIs) contribute to solving or at least mitigating many of the most pressing grand challenges. Similar to profit-oriented innovations, which are mainly developed by existing organizations and profit-oriented entrepreneurs, SIs are mainly developed and implemented by existing organizations and individual actors - social innovators. While much of the existing literature examines the needs of profit-oriented entrepreneurs and suggests entrepreneurial ecosystems as an adequate approach for satisfying profit-oriented entrepreneurs’ needs, little is known about the emerging needs of social innovators. By conducting an in-depth qualitative analysis of an exemplary territorial context based on 28 semistructured interviews and secondary data collection, this exploratory study aims to shed light on the emerging needs of social innovators. Furthermore, through an analysis of the identified needs, the study explores similarities and differences between the social innovation ecosystem and the entrepreneurial ecosystem. Thus, Isenberg’s entrepreneurial ecosystem model is leveraged for the development of a novel social innovation ecosystem model.

## Introduction

The world faces an increasing number of social and environmental problems, e.g., elderly unemployment, old-age poverty, migration, urbanization, and climate change. Additionally, the current COVID-19 pandemic poses challenges for billions of people, and solutions require collective and collaborative efforts from society as well as from a variety of actors around the globe (Bertello et al., 2021). The scholarly community has characterized these social and environmental problems as constituting *grand challenges* and is urgently seeking solutions for their mitigation (Colquitt & George, 2011). An increasing number of governments and policymakers are recognizing the relevance of these grand challenges and are including them in their agendas. On a global level, the United Nations set 17 sustainable development goals (SDGs) whose targets include most of the identified grand challenges (United Nations, 2015).

Although entrepreneurship is pivotal in tackling grand challenges, the profit orientation of entrepreneurial activity may be unsuitable for a large number of those challenges. Thus, a new approach is necessary for solving or at least mitigating many of the most pressing challenges that plague our society in the twenty-first century.

One approach, which has recently received increased attention from both the scholarly community and policymakers, involves social innovations (SI) (Akgüç, 2020; Bennett & McWhorter, 2019; de Wit et al., 2019; Gasparin et al., 2020). Despite the increased attention, the existing literature does not sufficiently consider and differentiate among the different SI actors developing and implementing SIs (Eichler & Schwarz, 2019). Two important actors (summarized in this paper as social innovators) are social entrepreneurs and nonprofit-oriented innovators who voluntarily develop SIs (de Wit et al., 2019; Westley & Antadze, 2010).

Much is known about the characteristics and needs of profit-oriented entrepreneurs (Acs et al., 2007; Duchek, 2018; Ratinho et al., 2020; Yusuf, 2010). The existing literature suggests entrepreneurial ecosystems as a suitable (if not the best) approach to satisfying those needs and fostering economic growth through profit-oriented entrepreneurial activity (Acs et al., 2018; Stam, 2018; Stam and van de Ven 2021). In recent years, the literature on the entrepreneurial ecosystem has continued to evolve in different directions. This paper, however, is based on one of the pioneering and most widely accepted entrepreneurial ecosystem frameworks (Al-Abri et al., 2018; Malecki, 2018; Song, 2019) as described in *Domains of the Entrepreneurship Ecosystem* by Isenberg (2011). We follow Cantner et al. (2020), who consider Isenberg’s six pillars (policy, markets, finance, culture, support, and human capital) as core elements of any entrepreneurial ecosystem and refer to them as the traditional entrepreneurial ecosystem.

Despite the growing knowledge on profit-oriented entrepreneurs, little is known about the characteristics of social innovators, their needs and how they can be supported in the development and implementation process of SIs. The scarce existing literature, which compares social innovators (particularly social entrepreneurs) with profit-oriented entrepreneurs, highlights various similarities and differences between the two actors (Abu-Saifan, 2012; Portales, 2019; Schneider, 2017). Similarities include the fact that both profit-oriented entrepreneurs and social innovators are individual actors. Therefore, successes or setbacks when developing and implementing an innovation are directly attributed to and experienced by the actor (e.g., compared to innovations developed and implemented by well-established (social or profit-oriented) organizations). Furthermore, profit-oriented entrepreneurs and social innovators are both initiative-taking, dedicated innovators characterized by high levels of persistence and commitment (Abu-Saifan, 2012). Due to the various similarities, ecosystems are expected to provide a suitable approach to meeting both the emerging needs of social innovators and fostering SI development and implementation. Additionally, similarities and overlap between an ecosystem for profit-oriented entrepreneurs and an ecosystem for social innovators could generate synergies, while differences between the two actors might lead to tensions.

Therefore, the purpose of this paper is to explore the characteristics of social innovators and their emerging needs in an exemplary region in which an SI ecosystem is still absent. Based on these empirical, qualitative data and by critically extending Isenberg’s model (2011), this study develops a novel model of social innovation ecosystems as partially overlapping with traditional entrepreneurial ecosystems and discusses possible synergies and tensions between these two ecosystems**.**

Thus, by applying a case study method, this exploratory study aims to identify whether incorporating social innovations into the entrepreneurial ecosystem context requires new research directions linking grand challenges to entrepreneurship.

## Theoretical underpinnings

### Social innovations (SIs) and their actors

While there is little doubt that profit-oriented entrepreneurship and innovations lead to the creation of economic wealth and are important for social and technological progress, the grand challenges confronting the world in the twenty-first century cannot be sufficiently solved by this approach. Instead, SIs are sought for the purpose of resolving (or at least mitigating) many of these grand challenges. SIs have been analysed by a broad spectrum of studies spanning a disparate range of fields and disciplines ranging from the IT field, e.g., the development of free open source software (Bhatt et al., 2016); the health sector, e.g., the study of dementia (Igarashi & Okada, 2015) and the housing and neighbourhood field (Blanco & León, 2017).

The novelty and wide range of academic fields analysing SIs are attributable to the lack of a common definition (Graddy-Reed & Feldman, 2015; Pellicer-Sifres et al., 2017). A previously conducted extensive definition analysis found that the following five elements best describe SIs: 1) a social need that must be addressed, (2) an innovative element such as a new approach, (3) implementation of a product or service, (4) improvement of a given situation, and (5) the development of new relationships and collaborations (Eichler & Schwarz, 2019). All five elements are contained in the definition by Murray et al. (2010).

As shown in Table 1, SIs are developed and implemented by different actors (Lundström et al., 2011). Similar to profit-oriented innovations (which can be developed and implemented by established firms as well as by entrepreneurs) (Schumpeter, 1942), SIs can be developed and implemented by established (social) organizations as well as by social entrepreneurs (Nandan et al., 2015; Westley & Antadze, 2010). Having been active in their corresponding markets for decades or even centuries, established social organizations are motivated by grand challenges and respond by creating innovative solutions. The SIs developed by established social organizations are either embedded within existing organizational forms or follow project-specific organizational forms. Social organizations tend to cross-finance their SI projects, and the risk of the responsible individual innovator(s) within the organization is low. An example illustrating the development and implementation of an SI by an existing social organization is the Caritas-founded Magdas Hotel Vienna. The most striking feature of the hotel, which was opened in 2015, is that it is mainly run by refugees and offers them the possibility of entering the labour market by attaining a job certification. As it is active in the social field, the organization was well aware of the challenges refugees face, and it had the necessary financial and human resources at its disposal to implement an SI. Because the building already belonged to the organization, it was available for use as part of an SI project. Additional well-known examples of established social organizations developing and implementing SIs are the Red Cross and the WWF (Christanell et al., 2019; Prasad, 2016; Windrum et al., 2016).

In addition, socially responsible businesses apply organizational forms that consider social aspects and focus on implementing SIs, e.g., through CSR, and thus they become SI actors (Mirvis et al., 2016; Szegedi et al., 2016). A well-known example is the Zweite Sparkasse (“second bank”), a program of the international Sparkasse Bank that supports people in economically challenging situations and offers them a way back to economic viability.

The starting conditions for social entrepreneurs are quite different. Even though exceptional social entrepreneurs have extensive market experience, many do not have any market experience at all and throw themselves in at the deep end. A social entrepreneur faces high individual risk as he or she acts individually and does not have the security of an established organization behind him or her. A common definition for social entrepreneurship (SE) is still absent, and the young field is characterized by competing definitions and frameworks (Lisetchi & Brancu, 2014). Martin and Osberg (2007) criticized the tendency in the contemporary literature and discourse to apply the term “SE” to a broad range of phenomena and instead developed a rigorous definition that distinguishes SE from social service provision and social activism. As derived from the term itself, a central aspect in SE is entrepreneurship. Abu-Saifan (2012) therefore reviewed and compared the profit-oriented entrepreneurship and SE literature and eventually offered a definition for SE that includes four central factors: social entrepreneurs are *mission driven*, and they *act entrepreneurially* within *entrepreneurially oriented organizations* that are *financially independent*. Other articles also aim to define SE by comparing it to profit-oriented entrepreneurship. The work of Santos (2012) is based on the holistic concept of value, which is critically assessed from the SE and profit entrepreneurship perspectives. He proposes defining SE as “*the pursuit of sustainable solutions to neglected problems with positive externalities*”. The added value of his work lies in his highlighting of the differences between value creation and value capture. The former is the clear focus of a social entrepreneur while the latter is the aim of a profit-oriented entrepreneur (Santos, 2012). Furthermore, and in contrast to a profit-oriented entrepreneur whose focus is on control, social entrepreneurs empower beneficiaries, users, partners and other actors outside their organizational boundaries. Thus, social entrepreneurs adopt a logic of empowerment (Santos, 2012). To understand the field of SE, it is essential to carefully consider the motivations and intentions of the entrepreneur him- or herself as much of the recent research does (Braga et al., 2014; Christopoulos & Vogl, 2015; Germak & Robinson, 2014; Hockerts, 2017; Renko, 2013). The role of individual motivations and intentions together with the contextual setting makes SE highly contestable and a challenge to measure (Huybrechts & Nicholls, 2012). It remains subject to interpretative assessment, which in practice (e.g., in determining the allocation of SE grants) is often conducted through advisory boards. Independent of how well a social entrepreneur is assessed, there is always the risk of post ante mission drift, i.e., an organizational change that neglects the original social mission (Cornforth, 2014; Ebrahim et al., 2014).

It should be emphasized that SE and SI are often incorrectly considered synonyms, and the terms are incorrectly used interchangeably (Domanski et al., 2017; Huybrechts & Nicholls, 2012; Westley & Antadze, 2010). Obviously, a social entrepreneur may develop and implement an SI, but SIs are also developed and implemented by other actors (Eichler & Schwarz, 2019; Lundström et al., 2011). Thus, social entrepreneurs are only one of a range of actors in the SI field, or, as stated by Phillips et al. (2015), “social entrepreneurs exist within a social innovation system”.

Another important SI actor is the nonprofit-oriented innovator, which refers to a volunteer without the intention of founding a business (de Wit et al., 2019; Westley & Antadze, 2010). Many nonprofit-oriented innovators do not have any market experience as they become active in the field due to unforeseen or even tragic events (e.g., a family member suffering from a disease, natural disaster). Through innovative solutions, the nonprofit-oriented innovator mitigates or even removes the negative consequences of the event and aims to create social impact for other people in similar conditions. A fundamental difference between a nonprofit-oriented innovator and a social entrepreneur is the motivation of financial return. While a nonprofit-oriented innovator usually has no intention of financial return (Baptista et al., 2019), a social entrepreneur has no intention of making a profit but intends to cover running expenses and allows himself/herself a personal salary (Santos, 2012).

Due to the different characteristics of SI actors highlighted above and the fact that social entrepreneurs and nonprofit-oriented innovators (summarized as social innovators) are individual actors who directly experience successes or setbacks when developing and implementing SIs, this study is focused on shedding light on the emerging needs of social innovators.

### Emerging needs of social innovators

The existing literature on the needs of social innovators is obscure and conflictual. Due to the abovementioned similarities between social innovators and profit-oriented innovators (Abu-Saifan, 2012; Portales, 2019; Schneider, 2017) and their expected commonalities, we use Isenberg’s (2011) traditional profit-oriented entrepreneurial ecosystem (Cantner et al., 2020) and its six pillars as a framework for characterizing the existing literature on the needs of social innovators.

#### Policy

can enhance the supply of and demand for SIs (Nesta, 2016). It can involve raising awareness about a social problem or activities that support SIs (Thompson et al., 2018; Toivonen, 2016). Policies that enhance SI education lay the groundwork for creating a fostering environment for SIs (Howorth et al., 2012; Kabbaj et al., 2016; Wals et al., 2015). The *Social Innovation Community* suggests five areas in which policymakers should support SIs: 1) funding, 2) supportive regulations and legal framework, 3) public procurement processes, 4) using public assets in socially innovative ways, and 5) raising awareness and building skills (Nesta, 2016). Other research stresses that in addition to policies for direct support, those enabling fiscal frameworks are also of high importance (Borzaga et al., 2013). In the past, social entrepreneurs often could only choose for-profit or nonprofit organizational forms, and recently, new types of legal identities suitable for the hybridity of social entrepreneurs (social and economic objective) have been established (Battilana et al., 2012). Policy can allow these new organizational forms to be further advanced and promoted.

Social innovators may receive specific financing at local, regional, national and international levels (e.g., the Structural Fund, the European Social Fund, the European Regional Development Fund, or the Social Entrepreneurship Fund in Austria (European Commission, 2013)). In other cases, SIs are supported by direct commissions, e.g., in the healthcare sector (Murray et al., 2010). Furthermore, social innovators can use new financing tools such as crowdfunding (Lehner, 2013; Rey-Martí et al., 2019). Many studies consider philanthropy an important financial source for SIs (Desa, 2010; Maclean et al., 2013; Moore et al., 2012). The type of finance differs depending on the actor. On the one hand, some actors pursue ventures that are financially self-sustaining (particularly social entrepreneurs). On the other hand, nonprofit-oriented innovators may establish voluntary associations that are primarily based on donations (Austin et al., 2012; Gandhi & Raina, 2018). In practice, the broad spectrum of financing possibilities often leads to the application of a so-called funding mix, a combination of earned revenue and non-revenue sources (Peattie & Morley, 2008). Due to their hybridity, social entrepreneurs have access to finance from both the for-profit and nonprofit sectors (Battilana et al., 2012). A hot topic in SI finance is impact measurement as suitable measures for evaluating the impact of SIs are not yet commonly agreed upon by scholars (Antadze & Westley, 2012; Geobey et al., 2012; Lee et al., 2019; Mihci, 2020). Impact measurement often represents a significant challenge for social innovators; e.g., the existing literature shows that social entrepreneurs fail to evaluate their social impact with sufficient regard to their social mission (Ormiston & Seymour, 2011).

#### Culture

is assumed to play a central role in the development and scaling process of SIs (Pratono & Sutanti, 2016; Westley & Antadze, 2010). For social innovators, a culture supporting entrepreneurship and innovations in general is needed as well as a culture of social sensibility. The former is characterized by aspects such as risk-taking, openness to new products or services or entrepreneurial drive (Cunha et al., 2015; Ionescu & Marga, 2015; O’Byrne et al., 2014). For the latter, ethics and aspects such as awareness and understanding of the social problem, altruistic behaviour, collective thinking and acting are essential (Cajaiba-Santana, 2014; Newth & Woods, 2014; Roundy, 2017a). Furthermore, SI development and implementation require cultures characterized by high levels of active societal participation and cooperation (e.g., within the neighbourhood or the community) (Alcaide et al., 2019; Domanski & Kaletka, 2018; Roundy, 2017a).

A central need for social innovators is the availability of suitable support. The problems faced by social innovators are often intensified by a wide range of engaged stakeholders or the nature of the problems themselves, leading to increased complexity (Lettice et al., 2010). A worldwide survey among more than 3,000 social entrepreneurs belonging to impact hubs showed that the top three support needs for social entrepreneurs are ‘feeling like part of a larger community’ (84%), ‘gaining visibility and credibility’ (76%) and ‘connecting to advisors and experts’ (73%) (Vandor & Leitner, 2018). Further important support for SIs, which has generally been neglected in the academic literature even though it has been well established in practice, are idea competitions and awards, e.g., the *European Social Innovation Competition* (Euopean Commission, 2020). SI idea competitions and awards help raise awareness of particular social issues and offer financial, material and training support to selected projects. In addition, they increase the visibility and credibility of winning projects. Depending on the SI, specific infrastructures and physical spaces may be required (Bloom & Dees, 2008) as well as project-specific support concerning legal aspects, access to networks or grant applications (Thomaz & Catalão-Lopes, 2019; Toivonen, 2016; Vandor & Leitner, 2018). Through specific programs (e.g., social accelerators), SI centres/hubs aim to satisfy many social innovator support needs (Alcaide et al., 2019; Domanski et al., 2019; Pandey et al., 2017).

The availability of sufficient and suitable human capital undoubtedly plays a fundamental role in social innovation. Estrin et al. (2016) analysed the role of human capital in social and profit-oriented entrepreneurship and showed that SE attracts different types of people than profit-oriented entrepreneurship. SE is particularly attractive to women, highly educated individuals, and unemployed individuals (Estrin et al., 2016). Concerning the required personal characteristics of social innovators, the existing literature highlights aspects such as persistence and endurance (Bikse et al., 2015; Braga et al., 2014; Konakll, 2015), which are necessary for handling setbacks in the SI development process. Furthermore, a certain level of case-specific knowledge is needed in developing and implementing SIs (Terziev & Arabska, 2017), and the nonlocal experience of the social innovator is considered to have a positive effect on the emergence of SIs (Kabbaj et al., 2016).

The markets in which social innovators are active often have unique characteristics. Social innovators often engage in activities characterized by market and governmental failures (Monllor, 2010; Pol & Ville, 2009). These markets are often less lucrative, and their customers and beneficiaries tend to be indistinguishable (Battilana et al., 2012). Furthermore, it is difficult to fully cover SI costs with revenues; therefore, many social innovators receive other forms of financial compensation (e.g., donations) (Gandhi & Raina, 2018). Additionally, as Pol and Ville (2009) highlight, SIs have features similar to public goods; therefore, in purely private markets, there will always be an undersupply of SIs unless governments intervene. Frequent SI markets are related to social and demographic changes or the need for environmental protection (Angelidou & Psaltoglou, 2017; Angelini et al., 2016; Groot & Dankbaar, 2014; Ludvig et al., 2019; Merkel, 2020). Other social innovators are active in niches of mainstream markets such as food or tourism and implement social or environmental-protective aspects in their innovative approaches (Alberio & Moralli, 2021; Malek & Costa, 2015; Pellicer-Sifres et al., 2017).

There are many ways in which the emerging needs of social innovators can be addressed and supported, e.g., grants, direct commissions, calls and prize money (Murray et al., 2010). From the entrepreneurship literature, however, we know that even if many elements have a fostering effect, in isolation, they are not sustainable, and the creation of corresponding ecosystems is suggested (Isenberg, 2010). In the following, we therefore briefly review the existing literature on SI ecosystems.

### Social innovation ecosystems

SI is not always clearly distinguished from the subfield of SE, and sometimes the two terms are even considered to be synonyms (Domanski et al., 2017; Huybrechts & Nicholls, 2012). Therefore, when reviewing the SI ecosystem literature, the literature on SE ecosystems also should be taken into consideration.

The literature on SI ecosystems is extremely scarce. Searching, e.g., in the Web of Science Database for ‘social* innov* ecosystem*’, leads to only four results that consider single important aspects of SI ecosystems such as social innovation labs (Alcaide et al., 2019; Domanski et al., 2019). Searching for ‘social* innov* system*’ leads to 100 results, of which only two articles (Rao-Nicholson et al., 2017; Surie & Groen, 2017) slightly broach the SI ecosystem topic.

Searching identically for ‘social* entrep* ecosystem*’ leads to 10 results that also address important single aspects of SI ecosystems (e.g. Bozhikin et al., 2019; Pandey et al., 2017; Thomaz & Catalão-Lopes, 2019).

Due to the limited number of studies identified in the Web of Science database, we extended our search using Google Scholar and applied a snowballing technique.

In the identified literature, the authors highlight the importance of social interaction in the formation of SI ecosystems. Thompson et al. (2018) stress that instead of top-down approaches, which often prevail in the implementation of entrepreneurial ecosystems for technological innovations, opportunities for engaging in social interaction are needed in the creation of SI ecosystems. Toivonen (2016) underscores the role of social interaction in SI, e.g., bringing together people or cultivating a shared culture of change making. Domanski et al. (2019) consider the importance of reconfiguring the interfaces of cross-sector cooperation and the role of a supportive infrastructure for SIs.

In a policy brief for the European Union, five pillars (resources, human capital, institutional robustness, social capital, market) are considered for creating an enabling ecosystem for social enterprises (Biggeri et al., 2017). The policy suggestions made in the policy brief are undoubtedly of high relevance on the EU level. However, they definitely must be broken down in subsequent steps to be applicable to the different EU countries as well as accommodate regional characteristics.

Only two papers have focused explicitly on ecosystems, although those papers exclusively consider social entrepreneurs as SI actors.

First, in a rather general paper in the *Stanford Social Innovation Review*, an ecosystem for social entrepreneurs is presented (Bloom & Dees, 2008). The suggested ecosystem consists of players such as resource providers or complementary organizations and environmental conditions, e.g., politics or infrastructure. Second, in a qualitative case study, the SE ecosystem of Morocco is analysed and mapped (Kabbaj et al., 2016).

Reviewing the scarce literature on SI and social entrepreneurial ecosystems makes clear that at a superordinate level, many of the supportive conditions and services are also addressed in other ecosystem strands, e.g., innovation ecosystems (Granstrand & Holgersson, 2019), business ecosystems (Mäkinen & Dedehayir, 2012) or entrepreneurial ecosystems (Audretsch & Belitski, 2017; Stam & Spigel, 2017). As explained in the introduction, due to the focus on social innovators and their similarities to profit-oriented entrepreneurs, the literature on entrepreneurial ecosystems is used as the starting point in this research and briefly reviewed in the following.

### Entrepreneurial ecosystems

The entrepreneurial ecosystem approach is based on an adaptation and modification of the biological concept of an ecosystem (Stam, 2015; Tansley, 1935). Researchers in the biological field define an ecosystem as ‘*an assemblage of organisms of different types (species, life forms) together with their abiotic environment in space and time*’ (Jax, 2006). The first sparks of the entrepreneurial ecosystem concept can be seen in Dubini (1989) and Van de Ven (1993). Cohen (2006) was one of the first to apply the entrepreneurial ecosystem approach, and in the last decade, it has gained attention in the areas of academics and policy making (Alvedalen & Boschma, 2017; Stam, 2015).

As with many new approaches in academics, there is no general agreement for defining entrepreneurial ecosystems (Alvedalen & Boschma, 2017). A well-known and often cited definition is from Isenberg (2010): ‘*the entrepreneurship ecosystem consists of a set of individual elements—such as leadership, culture, capital markets, and open-minded customers—that combine in complex ways. In isolation, each is conducive to entrepreneurship but insufficient to sustain it. … Together, however, these elements turbocharge venture creation and growth …’*.

The basic idea behind entrepreneurial ecosystems is the question of how to satisfy the needs of entrepreneurs and involved entrepreneurial ecosystem stakeholders (Isenberg, 2016). A recent literature review revealed that in most definitions, entrepreneurial ecosystems have geographically defined boundaries and include different interconnected actors and factors (Alvedalen & Boschma, 2017). To date, the entrepreneurial ecosystem literature mainly identifies several (sometimes very similar) factors fostering entrepreneurship (Alvedalen & Boschma, 2017) and presents implications for how to build an entrepreneurial ecosystem.

Among the most cited articles are ‘*How to start an entrepreneurial revolution*’, which was based on the Babson Entrepreneurship Ecosystem Project (also known as BEEP – Project) (Isenberg, 2010), and ‘*Domains of the Entrepreneurship Ecosystem*’, which contains a framework with six categories and 13 supporting factors (Isenberg, 2011). In the following years, other models were published such as the one by Stam (2015), which distinguished among four ontological layers (e.g., framework conditions vs. systemic conditions). In the past, entrepreneurial ecosystems were often considered at the country level and analysed correspondingly (Bernandezm & Mead, 2009; Stam, 2014; Voelker, 2012; Wessner, 2004). The recent research, however, has had a greater focus on geographically smaller regions such as entrepreneurial ecosystems in cities (Motoyama et al., 2016; Roundy, 2017b), in rural regions (Muñoz & Kimmitt, 2019) and on remote islands (Freitas & Kitson, 2018). Other recent studies have been dedicated to the development of entrepreneurial ecosystems in peripheral regions (McKague et al., 2017; Xu & Dobson, 2019). Despite these new research streams, the six core elements of the Isenberg model remain a dominant framework in entrepreneurial ecosystem studies (Cantner et al., 2020; Mack & Mayer, 2016; Maroufkhani et al., 2018). Due to their generality, they have been chosen as the basic, traditional entrepreneurial ecosystem model in this study.

## Method

### Methodological approach

Since the research on the emerging needs of social innovators and SI ecosystems is still in a nascent stage, an inductive research approach was chosen (Hyde, 2000; Mayring, 2000; Vaismoradi et al., 2013). More precisely, an exploratory qualitative research method was applied. We employed a single case study design (Baxter & Jack, 2008) and undertook field-based research by conducting an in-depth qualitative analysis based on a combination of expert interviews and secondary data collection. Qualitative field-based research is suitable for contributing to new theory (Eisenhardt, 1989; Glaser & Strauss, 1967; Strauss & Corbin, 1990).

The semistructured interviews (Drever, 1995; Whiting, 2008) began with a narrative element (Küsters, 2009) in which the interview partners were asked for the necessary conditions and services needed for the development and implementation of SIs. The subsequent questions were framed around the abovementioned six pillars adopted from the traditional entrepreneurial ecosystem. Particular attention was given to the unique conditions and services relevant in the development and implementation of SIs.

The first step was to identify and interview stakeholders and experts on the abovementioned six pillars. Then, social innovators and profit-oriented entrepreneurs were included in the designated sample. After that, a snowball sampling technique was applied to identify other social innovators and stakeholders (Coleman, 1958; Goodman, 1961).

### The case of Carinthia

The state of Carinthia (German: Kärnten) is located in the very south of Austria, bordering Slovenia and Italy. It currently has approximately 560,000 residents and a surface area of approximately 10,000 km^2^. The capital and largest city of Carinthia is Klagenfurt (100,000 inhabitants), followed by Villach (60,000 inhabitants) and Wolfsberg (25,000 inhabitants).

Carinthia faces many grand challenges such as an outflow of the (young) population (Stockhammer, 2018) and the third-highest negative birth balance in Austria (Wirtschaftskammer, 2017). Together, these two factors are responsible for making Carinthia the only Austrian state with a population decline (Wirtschaftskammer, 2019). Related to the population decline is the problem of its ageing society, which is much more intense than in the rest of Austria (Fercher, 2015). In addition, as it is located in the Alps, Carinthia faces an increasing number of environmental and climate change-related challenges. Based on these data and characteristics, Carinthia is a suitable example of a region in need of SIs.

The particular situation of Carinthia and its economic challenges were the basis for the founding of a business incubator in 2002. Driven by the region’s university and the business incubator, an ecosystem for profit-oriented entrepreneurs emerged. Today, this entrepreneurial ecosystem is well established and has a strong focus on technology. Undoubtedly, the ecosystem for profit-oriented entrepreneurs had and continues to have a positive impact on the region’s wealth. However, it can be observed that the prevailing ecosystem for profit-oriented entrepreneurs does not support social innovators sufficiently.

After conducting 28 interviews (see Table 2) lasting between 1 and 1.5 h each, saturation was reached from two perspectives (Fusch & Ness, 2015; Glaser & Strauss, 1967). First, no new information on the emerging needs of social innovators was obtained by conducting further interviews. Second, at the end of each interview, the interview partner was asked to suggest additional interviewees to obtain a better understanding of the emerging needs of social innovators. Saturation was reached when all suggested additional interviewees were already contained in the sample.

**Table 1:**
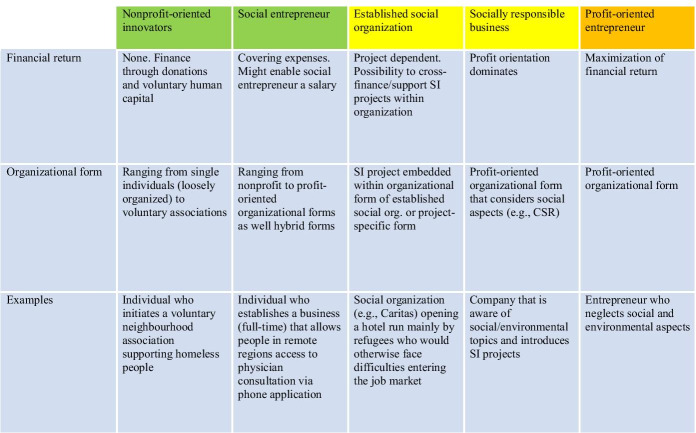
Actors developing and implementing SIs. Source: own creation

**Table 2:** Overview of interview partners

Interview partner	# of interviews	Abbr	Functions + Fields	Additional data
**Social Innovators**	**11**			
Social Entrepreneurs	6	SE 1–6	Health and care taking, responsible consumption, environment protection, income perspectives for poverty affected people	Homepages, public interviews, social media, video pitches, observations, informal talks
Nonprofit-oriented Innovators	5	NPI 1–5	Responsible consumption, health, education, mobility	Homepages, public interviews, social media, video pitches, observations, informal talks
**Profit-oriented Entrepreneurs**	**3**	PE	Technological water metres and waste management, drones, formwork (concrete)	Homepage, public interviews, social media
**Stakeholders**	**14**			
Political	4	P 1–4	Regional minister, mayors	Public interviews, newspaper articles
Finance	2	F 1–2	Director and manager of financial support office for regional development	Homepages, information on supporting projects
Culture	2	C 1–2	Professors of cultural science and cultural studies	Publications
Support	2	S 1–2	Director and manager of regional incubator	Homepages, social media
Human Capital	1	HC 1	Manager unemployment agency	Homepages
Markets	3	M 1–3	Director and manager of social organizations, social innovation project consultant	Social media, newspaper articles

**Table 3:**
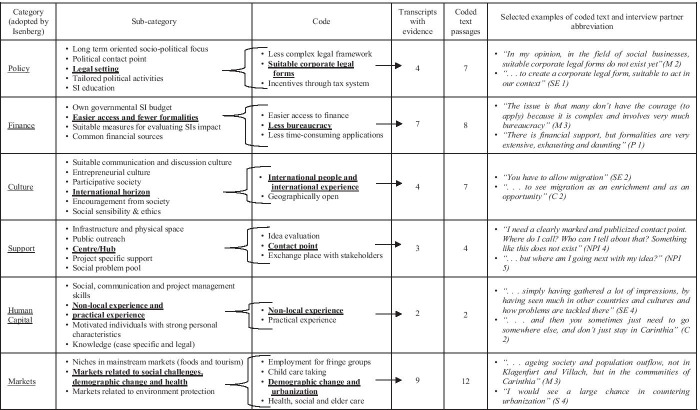
Illustration of the coding process on selected subcategories and codes

The interviews were transcribed and coded using the qualitative data analysis software NVIVO (Bazeley & Jackson, 2013). Coded text passages were classified according to categories and subcategories. Following a direct content analysis approach (Hsieh & Shannon, 2005), we defined the categories before starting the analysis (six pillars) as well as during the analysis (subcategories summarizing diverse opinions). In the following step, the multiple subcategories were streamlined and condensed into fewer subcategories following Saldana’s (2013) approach. Table 3 illustrates the coding process and depicts a portion of the coded interview transcripts.

Additionally, to carry out the in-depth qualitative analysis and thus to increase the validity of the identified subcategories, a variety of collected secondary data were used for triangulation (Carter et al., 2014; Jonsen & Jehn, 2009). Of particular value were video pitches, observations, and social media of social innovators and profit-oriented innovators as well as newspaper articles, homepages, and recorded public interviews from different stakeholders (see Table 2).

## Results: emerging needs of social innovators

As mentioned in the methodology, Isenberg’s six pillars were used as a framework for the analysis. The 27 emerging needs of social innovators identified in this exploratory study are represented following the same structure.

### Policy

The most crucial policy need that emerged in this study is a long-term oriented socio-political focus. This need influences all other policy elements as it contains answers to the fundamental question ‘where do we want to move as a society’ (M 1). To address this need, it is necessary that the ‘political shortcoming of thinking in short intervals’ (F 1) is eliminated and that the mindsets of politicians privilege social aspects. This issue is noted by M 3, who stresses that ‘*SI needs to be put on political agendas*’, and by SE 1, who seeks a ‘*clear political commitment to the topic*’. More specifically, F 2 highlighted the issue that ‘*politics is short-term oriented … and in order to create such conditions I need longer-terms*’. It is a straightforward concept that a supportive long-term oriented socio-political focus also includes politicians’ awareness of social challenges and the potential of SI for mitigating them (NPI 3, SE 5). The study also showed the necessity of having a political contact point. Several interview partners mentioned the importance of having a point of contact (NPI 3, SE 2). P 1 considers herself a political contact point-person for social innovators: ‘*without any doubt, he comes to me*’. A logical consequence of a long-term oriented socio-political focus and the introduction of a political contact point are tailored political activities that support SIs. They include efforts to raise awareness of the social problem by ‘*being present and talking about the topic*’ (NPI 1) and highlight the SI itself by ‘*staying behind the innovation and bringing it into media*’ (NPI 1). The remaining political activities are rather heterogeneous and include, e.g.*, ‘calls by the regional government for certain topics*’ (S 2) or better cooperation with society; thus, ‘*the ideas come from society and politicians need to absorb them*’ (NPI 5). Interviewed political stakeholders highlighted their engagement in a large set of different supportive activities such as raising awareness of SIs (P 3, 4), providing financial support (P 3, 4) and fostering civil involvement in activities (P 2). Five interview partners mentioned the need for policymakers to introduce SI education. M 3 explained that education must deal with ‘societal problems, how they emerge and how they can be avoided’ or at least how they can be solved and mitigated (M 3, NPI 2). Thus, education should include topics such as social change because ‘we as a society are in a [process of] continuous change’ (M 1). Several interview partners (e.g., NPI 1, M 1) stressed that the education aspect should be integrated ‘from kindergarten till university’ (M 1). Regarding the legal setting, it is interesting to observe that, independent of the role of the interview partner, a more suitable and less complex legal framework is seen as crucial for fostering SIs. While PE 2 highlights that, in general, ‘*for any type of innovation, it would be easier if the complex regulations would be eliminated*’, SE 1 is more specific and asks for suitable corporate forms. A further condition often mentioned is incentives through the tax system. This could be, for example, a lower tax for repair service (S 1), a lower tax rate for start-ups (SE 3), or tax deductions when achieving social impact (SE 1).

### Financing

Many interview partners explicitly stressed that easier access and fewer formalities for financial support are needed. More precisely, NPI 5 mentioned that regardless of whether the funding is obtained through ‘*... crowdfunding, grants, or whatever, the access to it needs to be simplified*’. Indeed, in almost every interview, there were hints that social innovators need financing with fewer formalities such as less bureaucracy (e.g., NPI 2, P 3, P 1); easier access to money (e.g., SE 5, NPI 5, M 3); or less time-consuming applications (SE 4). F 2 even describes ‘*strange regulations and laws*’ and a ‘*jungle of support*’. Another frequently mentioned argument dealt with suitable measures for evaluating SI impact (e.g., NPI 1, SE 1, SE 2) and the need for wise decisions about how funds are distributed (e.g., SE 2, NPI 2, PE 3). F 1 confirms that no specific financial support for SIs has been offered until now and that support for (voluntary) associations is currently decreasing (F 1). Regarding the type of finance, common financial sources were mentioned such as bank credit (SE 6); grants (S 1); financing from existing enterprises (SE 5); crowdfunding (NPI 1, PE 3); and investors (NPI 1). Furthermore, three social innovators explicitly requested their own governmental SI budget for the financial support of SIs (NPI 2, SE 1, SE 2).

### Culture

A suitable communication and discussion culture means that ‘*people have the courage to express their opinion*’ (NPI 5) but respect each other; as highlighted by C 2: ‘*learning how to debate*’ is needed. PE 2 noted that ‘*if people talk, crazy ideas emerge that can be implemented*’. The media plays a fundamental role in the emergence of a suitable communication and discussion culture (P 2). According to the interview partners, in an SI culture, it is important that society encourage social innovators and SI initiatives (e.g., SE 5, S 1 PE 1, P 1). In that context, society ‘*recognizes achievements and admires that someone dares to do something*’ (SE 4). By ‘empowering people in what they are doing’ (M 3), the social innovator eventually begins to believe in himself or herself (M 3). In an era of globalization, an international horizon is fundamental, as highlighted in seven interviews. Such an international horizon can be achieved if society can “move out of its comfort zone”, connect with other cultures (NPI 3) and accept that SIs do not exclusively come from the inside but may also come from the outside (PE 1). Furthermore, it is crucial to ‘*accept immigration*’ (SE 2) because immigrants have different perspectives, speak different languages and come from different cultural backgrounds (S 1). Thus, it is necessary ‘*to see migration as an enrichment and an opportunity’* (C 2). Therefore, an internationally open culture with a cross-border mindset is needed (F 1). Openness is also the dominating element in the entrepreneurial culture category. In 16 interviews, interviewees described an open culture as essential for the development and implementation of SIs. They explained that this may be the case because such a culture is needed for a society to be ‘*open [to] change*’ (S 1) and thus willing to ‘*try something out and accept something innovative*’ (SE 4). Such a society would then face ‘*little fear of something new*’ (PE 2). Concerning the need for a prevailing participative society, interview partners highlighted that the participation and involvement of different stakeholder groups in the development of SIs is essential (M 3). Such groups were said to include different age groups (S 2) and those affected by a given problem (M 1). Another important aspect concerning culture is social sensibility and ethics. In addition to ethics (SE 2), this aspect includes values and norms (C 2) and the local environment (and its protection) (M 3). Furthermore, the interviews showed that social cohesion (e.g., NPI 1, NPI 2, NPI 5); awareness of (social) problems (SE 2, F 1, F 2) and ‘*a sensitivity for certain problems*’ (C 1) are crucial for the development and implementation of social innovations. Furthermore, social sensibility includes an active participating culture that is understood as having a ‘*willingness to participate, even if it does not give financial remuneration*’ (SE 1) and a society with a ‘*friendly, supporting atmosphere*’ (M 3).

### Support

The support category contains assistance in public outreach such as raising awareness about the social problem (e.g., SE 2, NPI 2) and giving visibility to successful social innovators (e.g., SE 4, F 1). The media plays a particular supportive role in this regard; as PE 1 stressed, ‘*media could foster SIs, be it newspapers, TV or internet*’. Another fundamental need of social innovators is the availability of suitable infrastructure and physical space. The availability of high-speed internet in peripheral regions (S 2, C 1) and physical places, such as free or cheap offices (SE 3, PE 1) or coworking places (HC 1, P 2), are seen as fundamental. Additionally, project specific support is necessary. Support in accessing and establishing project-specific networks was described as fundamental by nine interview partners, who stated that such support could be fostered by events or workshops. Other noted types of project-specific support included legal and insurance advice (SE 6, NPI 5), assistance with grant applications (SE 6, M 3), and the assessment and development of business plans (SE 6).

The interview partners highlighted the need for a centre/hub, a physical place that serves as a contact and information point that is well promoted and easily contacted (SE 5, NPI 4, NPI 5). Despite the wide range of related terms and synonyms mentioned in the interviews such as focal point, place to go, platform, drop-in centre or ‘*an own SI centre*’ (SE 6), from the context, it is clear that the respective interview partners were in agreement with regard to the need for such a centre/hub. A place where the SI idea can be presented and evaluated (e.g., NPI 3, NPI 4, SE 4, P 4) before the social innovator is directed to the corresponding stakeholder is needed. Therefore, such a centre should be ‘*familiar with the entire setting*’ (M 1) and ‘*in exchange with all relevant stakeholders*’ (S 1). SE 4 shared his own experience on this issue as, for him, it had initially been very unclear where to go; thus ‘*a central, single contact point instead of ten is needed*’. A unique support need of social innovators is the availability of a social problem pool (NPI 3).

### Human capital

Four elements stood out in the human capital category. The element that was mentioned most involved the type of strong personal characteristics that affect motivation. Such characteristics were described as important, ‘*particularly the motivation to become active*’, as noted by (SE 4). The importance of persistence was also mentioned, which can mean both ‘*not being dissuadable*’ (M 3) and ‘*an immensely high level of endurance*’ (NPI 4). Furthermore, social innovators require a high level of courage and openness (e.g., SE 4, SE 5, NPI 1). In addition to the personal characteristics discussed above, we found evidence for the necessity of different social, communication and project management skills. More precisely, social competencies (SE 4), team-working skills (SE 2), and cooperating skills (M 3) were highlighted. HC 1 stressed that ‘*you work in pairs of two or three on those ideas*’ (HC 1). Other skills described as important were communication skills to market an idea (SE 5, P 3) or to convince others (P 1) as well project management skills (M 1, F 2). The third human capital category, specific knowledge, mainly contains case-specific knowledge (e.g., NPI 2, SE 3) and legal knowledge, e.g.*, ‘How do I make my tax declaration*’ (SE 2). With regard to experience, the interview partners mainly stressed the role of nonlocal experience outside the region to understand ‘*how problems are solved there*’ (SE 4) and practical experience (P 2, F 2).

### Markets

It was not possible to identify the general characteristics of SI markets due to the strong differences depending on the specific SI product or service. However, three market categories were identified that offer large potential for social innovators in the case study region. The first market category is markets related to social and demographic change; 50% of the interview partners, including the market experts, considered the health, social care, and care-taking field to be a market with considerable potential for SIs. There is also large potential for SIs in topics related to urbanization such as ‘*countering urbanization*’ (SE 4). The second market category is markets dealing with environmental protection, which offer large potential for SIs and include, e.g., a reduction of plastic (NPI 1) or in CO2 emissions (S 2). The third market category, niches in mainstream markets, offers many possibilities for social innovators. In the case study region, these niches include the food market, e.g., by ‘*saving groceries and thereafter processing them*’ (NPI 3) or the provision of regional food directly from the producer (NPI 5). Another niche market with potential for SIs is that of tourism, particularly health tourism and sustainable tourism (SE 2, PE 2).

## Discussion

The analysis of the identified emerging needs of social innovators confirms the anticipated assumption of fundamental differences and similarities between social innovators and profit-oriented entrepreneurs. Sixteen identified needs of social innovators are also found to be relevant for profit entrepreneurs and are considered in the corresponding literature. Eleven identified needs are found to be social innovator-specific.

**Fig. 1:**
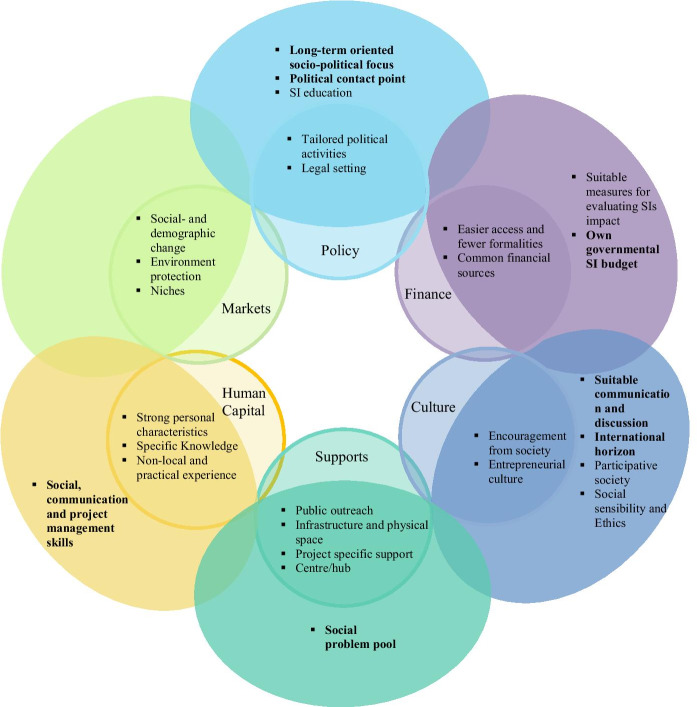
Social Innovation Ecosystem Model. The six inner circles represent the traditional entrepreneurial ecosystem; the six exterior circles represent the social innovation ecosystem. Emerging needs largely neglected in the literature are in bold

Based on the findings, a social innovation ecosystem model was developed (six exterior circles) partially overlapping with the traditional entrepreneurial ecosystem (six interior circles) (Fig. 1). Each of the six pillars is elaborated in the following with a focus on emerging needs that represent an update to the traditional entrepreneurial ecosystem. The emerging needs of social innovators, which are largely neglected in the literature, are highlighted in bold in the model.

### Policy

Largely neglected in the SI literature and not contained in the traditional entrepreneurial ecosystem is the need for a long-term oriented socio-political focus. An explanation for the absence of a long-term-oriented socio-political focus in the traditional entrepreneurial ecosystem might be that profit-oriented entrepreneurial activity focuses exclusively on economic wealth creation. In contrast, SI focuses on total wealth creation composed of social and economic wealth (Zahra et al., 2009). Therefore, the economic wealth creation of profit-oriented entrepreneurs tends to occur faster and can be more easily measured than the total wealth created by SIs. Even though there is occasional political support fostering SIs, such support is often short-term-oriented and legislature-dependent. As SI output does not always occur in predictable time frames (Antadze & Westley, 2012), and it may take time to recognize its effects (particularly with regard to positive effects on the environment), short-term thinking in politics is a severe problem for nascent SIs. Politicians often prefer to adopt policies or support specific projects whose effects occur within the current legislative period and thereby increase their chances of re-election. Therefore, SIs are often not the focus in political decision-making. Moreover, even if a nascent social innovator and his or her project is supported by a politician (or political party), there is the risk that a newly elected politician might no longer support the project as its social impact is not yet evident on a large scale. While a long-term-oriented sociopolitical focus involves many aspects, a possible starting point might be to consider social impact in tax systems. This could either be done by offering general tax benefits for social enterprises (Vasin et al., 2017) or by lowering tax rates on services and products with a positive social and/or environmental impact that are not entirely cost-covering.

A political contact point represents the second policy need of social innovators, which is largely neglected in the SI literature and is not contained in the traditional entrepreneurial ecosystem. A possible explanation of why social innovators express the need to have a political contact point is that their SIs can often be found in fields such as health care and elder care, which have traditionally been managed by governments and public administrations. In such fields, cooperation and exchange with politicians and public administrations are essential for multiple reasons. On the one hand, these fields are characterized by strict regulations; on the other hand, social innovators might overtake a task from public administration; thus, the question of financial compensation arises. Therefore, before implementing an SI in such a field, extensive exchanges between politicians and the social innovator are necessary. The earlier the social innovator can establish cooperation with politicians, the earlier he or she will be aware of the circumstances in the field and thereby be able to address the required regulations in the SI. Additionally, for many SIs, customer-beneficiary relations are complex, and the beneficiary might not be the one making the payment (Battilana et al., 2012; Vandor et al., 2015). Without knowing the situation for profit-oriented entrepreneurs, the given study showed that social innovators face difficulties getting in touch with politicians. While the interviewed mayor of a small village mentioned that social innovators consider her a political contact point and meet with her, it is evident that it is more difficult to get into touch with politicians in larger cities. The political contact point should serve as the point person who can send the social innovator to the appropriate responsible political offices and thereby takes on a mediation role.

Without a doubt, policy fostering the introduction of SI education requires an update on the traditional entrepreneurial ecosystem. SI education, however, is not entirely new as many different studies address the topic. In practice, however, despite its increasing attention (Howorth et al., 2012; Kabbaj et al., 2016; Wals et al., 2015), SI education remains a rarity. This becomes even more evident when compared to entrepreneurship education, which comparatively has become a fixed component in many universities and even high schools (Hägg & Gabrielsson, 2019; Nabi et al., 2017). Unlike entrepreneurship education, which focuses on identifying market gaps, SI education must first stimulate the awareness and importance of prevailing social problems. Second, instead of suggesting the founding of a profit-oriented business for implementing business ideas, SI education must present students with the different ways of implementing SIs (e.g., hybrid business forms, voluntarily associations). Bethany et al. (2015) suggest considering transformational, critical reflection and place-based learning and going beyond the standard curriculum when teaching SI. Furthermore, we suggest including ethics and social responsibility (Thorsteinsson, 2013, 2014) in SI education.

The other two identified policy needs, tailored political activities and legal setting, are relevant for social innovators and profit-oriented entrepreneurs. They therefore represent an overlap of the two ecosystems.

### Financing

Concerning the finance pillar, two identified needs represent an update on the traditional entrepreneurial ecosystem. First, suitable measures for evaluating SI impact are needed due to the different characteristics of innovations by profit-oriented entrepreneurs and innovations by social innovators. Although the literature considers this need and generally agrees that the existing measures from the profit-oriented entrepreneurial field are unsuitable for evaluating SIs (Geobey et al., 2012; Lee et al., 2019; Mihci, 2020), generally agreed upon suitable measures for evaluating SIs are still absent. It might be even questionable whether perfect measures for evaluating SI impact can ever be developed as SIs are very heterogonous. Second, the introduction of a governmental budget for SI, which is not considered in the literature, would offer social innovators the possibility of more easily accessible financing. Although governments currently occasionally financially support SIs by providing financial means, this financial support is mostly project-related, and its availability depends on the current political composition (see the need for a long-term-oriented socio-political focus). This issue can be overcome by creating a permanent governmental SI budget. Furthermore, a governmental SI budget offers the possibility of a long-term-oriented promotion of the particular issue. With the knowledge that financial support for SI is not available only in the short-term but that, rather, with the inception of a governmental budget, such support would remain a permanent feature, more potential future social innovators would be encouraged to dedicate effort to their ideas. Last, due to the special customer-beneficiary relationships of certain SIs (Battilana et al., 2012) and their nonexcludable product or service characteristics (e.g., positive effects on health or environment), dedicated governmental financial support is necessary for many SIs. Such dedicated governmental financial support could be offered – at least when SI projects are still in their nascent and early stages.

The remaining two emerging finance needs of social innovators, easier access and fewer formalities for financing and the availability of common financial sources, are also applicable to profit-oriented entrepreneurs and are addressed in several studies. Not all profit-oriented entrepreneurs, and not all social innovators have high education levels, and preparing a complex founding request often represents a large hurdle. Therefore, more easily accessible financing is needed as well as assistance in writing foundation requests.

### Culture

The culture pillar represents the field where most (four) updates to the traditional entrepreneurial ecosystem are needed.

First, identified as necessary for SI development and implementation but still largely neglected in the SI literature is the need for a suitable communication and discussion culture. As stated in the definition, SIs aim to improve capabilities and relationships (Eichler & Schwarz, 2019; Murray et al., 2010); therefore, it is a common matter rather than a single combat. Furthermore, solutions to social or environmental challenges often emerge through the discussion among many people. Once an innovative idea for solving existing challenges emerges, social innovators focus on empowerment while profit-oriented entrepreneurs focus on control (Santos, 2012). More precisely, social innovators aim to empower many other actors (e.g., beneficiaries, users, partners), and in certain cases (particularly for SIs implemented by the creation of a volunteer association), the number of involved individuals can easily make up dozens of people. Therefore, a prevailing suitable communication and discussion culture is essential. Obviously, also a profit-oriented entrepreneur usually cooperates with his or her partners. However, in such cooperation, the profit-oriented entrepreneur always aims to maintain control over his or her innovative idea and to keep power relations clear. It is therefore apparent that the more people there are involved in the development, implementation, and scaling of an innovation, the more heterogeneous the opinions will be. Therefore, communication and discussion must be characterized on the one hand by a high level of respect; on the other hand, they must also involve determination and decisiveness.

Second, there is an emerging need for a culture with an international horizon, and its absence in the SI literature is surprising, particularly considering the increasing attention to the scaling of SIs (Voltan, 2017; Voltan & De Fuentes, 2016; Westley & Antadze, 2010). For SIs, an international horizon is needed from the perspectives of both current and future social innovators as well as from that of society, which is confronted by SIs in daily life. In times of globalization, most of the grand challenges are not country-specific but exist (with different intensities) worldwide. It can even be said that a majority of the existing challenges are not contained within any borders (e.g., poverty, environment protection) and, as stressed through the 17 SDGs, they require global effort (United Nations, 2015). Unlike the profit-oriented entrepreneurial field, where the entrepreneur often thinks and acts globally from the very beginning, in the SI field, the social innovator mostly limits himself or herself to the local environment. The term “born global firm” is often used as a synonym for technological profit-oriented entrepreneurs, while born global SIs are hard to find. Obviously, it is already positive if society is aware of the surrounding social problems. However, by reflecting on problems that may exist in similar ways in other regions and countries, potential innovative ideas would have a much more substantial impact. Thus, considering international product characteristics in the SI process is helpful as locally developed SI might also work in other regions and countries. In other words, an international horizon multiplies the potential market size and the created social impact. Furthermore, an international horizon is needed as important stakeholders for developing an SI might be located in different countries. In addition to the needed international horizon of current and future social innovators, it is also important that society and its prevailing culture become more internationally open. This means that society would be able to accept that an entire SI solving a local problem may come from the other side of the world. It remains unclear why societies where technological products and services are sold worldwide close themselves to SI approaches from foreign countries. Possible explanations might include the fact that SIs can often be found in sensitive fields or require changes in long traditions.

Third, a high level of participative society is necessary for SI development and implementation. However, the great importance of a participative society is not new in the field of SI (Cajaiba-Santana, 2014; Roundy, 2017a), and it has received another boost of attention during the COVID-19 pandemic (Andion, 2020; Bertello et al., 2021; Cattivelli & Rusciano, 2020). If society is stubborn and does not participate at least by purchasing an innovative product or service, every innovative idea fails. However, for SIs, a participative society may play a fundamental role not only at the purchasing stage but also in the entire development and implementation process. More precisely, a participative society is crucial for the development of innovative ideas to prevailing societal problems, and the development of SIs usually involves many different stakeholders as well as those affected by the problem. Once an SI is ready for implementation, societal participation is crucial in offering the SI product or service since financial compensation for individuals’ efforts is secondary.

Fourth, a substantial update to the traditional entrepreneurial ecosystem model is needed concerning the role of social sensibility and ethics, which is well acknowledged in the SI literature. While traditional entrepreneurial ecosystems focus on aspects such as tolerance of risk, ambition or entrepreneurial drive (Isenberg, 2010), for SIs, questions such as “environmental impact of the product”, “CO^2^ footprint”, and “do I create social impact by purchasing a certain product?” play a fundamental role. Reflecting on such aspects and considering them in the purchasing process makes society aware of the prevailing problems and facilitates the public’s understanding of them. However, social sensibility and ethics are crucial not only for society but also for the social innovator himself or herself. For example, the social innovator often needs a high level of social sensibility (which may also include empathy) since the addressed problem involves sensitive topics and/or often includes vulnerable groups, e.g., the unemployed, the elderly, or people suffering from dementia (Grimm et al., 2013; Igarashi & Okada, 2015; Ims & Zsolnai, 2014). Furthermore, social innovators often become active due to their religion or other ethical obligations (Dodd & Gotsis, 2007; Zahra et al., 2009).

Encouragement from society is crucial for profit-oriented entrepreneurs and social innovators and thus represents one of the two cultural overlappings between the traditional entrepreneurial ecosystem and the SI ecosystem. Encouragement and support from society play an important role when experiencing setbacks or lacking urgently needed resources (Hopp & Stephan, 2012). Furthermore, many social innovators invest enormous effort in their innovation without ever receiving any/much financial return. Therefore, societal encouragement and appreciation might provide a vital motivational boost. As broached in the interviews, adequate media coverage plays a key role in achieving a culture that encourages social innovators.

The need for a prevailing entrepreneurial culture is the second cultural overlap. Regardless of whether a project is profit-oriented or socially oriented, developing and implementing an innovation always involves high risk (Kuckertz et al., 2020; Vereshchagina & Hopenhayn, 2009). Therefore, a culture in which individuals are willing to take risks must be stimulated, e.g., through corresponding entrepreneurial education (Rina et al., 2018). However, the entrepreneurial culture aspect not only focuses on the innovator himself or herself but also involves how society deals with failure. Furthermore, it is crucial that society be open to new products and services because the best innovation is purposeless if it is not acquired and used.

### Support

The need for a social problem pool appears to be unique to the field of SIs and requires a strong update to the traditional entrepreneurial ecosystem. Even though social problems are the core of SI, in the literature, this type of pool is not yet addressed. By pooling social problems, such a support institution would take over many important tasks. First, it would raise awareness about certain social problems. Second, it could elaborate the relevance and complexity of those problems. Third, it acts as a contact point for prosocial motivated individuals who do not have a problem much less an innovative idea for solving the problem in mind. For example, many pensioners are still in good shape and prosocially motivated when they retire. However, because they have spent most of their lives focusing on their jobs, they are often unaware of the current problems in society when they retire. By classifying and promoting some social issues and bringing together individuals who are interested in working on innovative solutions to problems, such an institution would also contain an important networking function.

The four additional identified needs are also relevant for profit-oriented entrepreneurs and overlap with the traditional entrepreneurial ecosystem.

First, both social innovators and profit-oriented entrepreneurs need support in terms of **public outreach** (Lorrain & Laferté, 2006; Vandor & Leitner, 2018). In practice, public outreach can be supported through events, competitions, shows, and diverse media contributions.

Second, infrastructure and physical space are needed. In the broader sense, infrastructure contains high-speed internet. Physical space is always offered in more cities in the form of coworking spaces. In addition to offering offices for rent, coworking spaces also play a crucial role in establishing networks among social innovators and/or profit-oriented entrepreneurs.

Third, regardless of whether they are socially or profit-oriented, many innovations are rather complex and require project-specific support. Such support also involves assistance in accessing corresponding networks.

Fourth, the need for supporting centres/hubs is not only addressed in the traditional entrepreneurial ecosystem literature but also in the SI literature (Alcaide et al., 2019; Domanski et al., 2019; Pandey et al., 2017). Such a centre/hub is differentiated from the social problem pool introduced above as it is necessary to have a rough business idea in mind when approaching such a supportive institution.

### Human capital

Individuals with strong social, communication and project management skills are crucial for SI emergence and thus represent an update to the traditional entrepreneurial ecosystem. Despite its importance, this aspect is still largely neglected in the SI literature. SIs typically emerge through discussion or collaboration among many individuals, and SIs are often implemented by teams, so social and communication skills are essential. Furthermore, practical social and communication skills are necessary for finding employees and associates as well as for offering SI (highlighting the social impact) to society. An SI may involve vulnerable groups of people who have endured difficult times (Grimm et al., 2013; Igarashi & Okada, 2015; Ims & Zsolnai, 2014). Thus, in such cases, more sensitive communication is required. Last, project management skills require attention when updating the traditional entrepreneurial ecosystem model. Due to the different characteristics of SI projects, it is unlikely that the same skills will be suitable for profit-oriented entrepreneurial projects.

Moreover, this study identified three emerging human capital needs that are also relevant for profit-oriented entrepreneurs and represent overlappings with the traditional entrepreneurial ecosystem.

First, individuals with strong personal characteristics must have the courage to become active and initiate an innovation. The ensuing process requires a high level of endurance as the development and implementation of any innovation involves setbacks.

Second, it is evident that in many cases, project-specific knowledge is needed (e.g., innovations that involve high-end technology or that are embedded in difficult legal settings).

Third, individuals with nonlocal and practical experience are fundamental. The “hands-on” mentality often mentioned in a profit-oriented entrepreneurial context is also crucial for SI development and implementation. Practical experience, ideally related to the focal social problem, plays a key role throughout the SI development process (Asante et al., 2020). Individuals with experience from outside a region may boost the entire ecosystem, either by sharing their experience with contributors to existing SI projects or by initiating an SI themselves.

### Markets

The market pillar is the only one that does not require updates to the traditional entrepreneurial ecosystem, as all three identified markets offer large potential to both social innovators and profit-oriented entrepreneurs. Markets related to social and demographic change become more important due to, e.g., an ageing society, modern family structures, or urbanization. While profit-oriented entrepreneurs identify lucrative market gaps related to these phenomena, social innovators aim to solve or mitigate grand challenges in a given field. Opportunities related to environmental protection emerge as the effects of climate change intensify and become more visible. Profit-oriented entrepreneurs develop and implement profitable innovations with a positive effect on the environment that are not seldom financially supported by governments or public administrations. Additionally, social innovators develop and implement innovations with a positive environmental effect. Furthermore, they are engaged in innovations that raise awareness about environmental challenges. From two different perspectives, market niches offer the potential for profit-oriented entrepreneurs and social innovators. Profit-oriented entrepreneurs may opt for a niche as it could allow them to apply a differentiation strategy. In contrast, social innovators may focus on a niche that is not profitable and therefore neglected by entrepreneurs and businesses. Even though no further market-related needs could be identified in this study, it is likely that certain elements such as early adopters, reference customers, or distribution channels contained in the traditional entrepreneurial ecosystem also apply for social innovators.

### Synergies and tensions in leveraging the traditional entrepreneurial ecosystem

Leveraging the traditional entrepreneurial ecosystem by the eleven identified needs of social innovators offers the possibility that both profit-oriented entrepreneurs and social innovators will eventually be supported by the same ecosystem. Such an approach is expected to lead to synergies and entails several positive aspects. First, as mentioned above, to battle the grand challenges faced by society, and to reach the SDGs by 2030 (United Nations, 2015), new types of innovation and entrepreneurial activities are needed. Policy makers thus often face the decision of whether to stimulate profit-oriented entrepreneurs towards a more social orientation or to opt directly for an SI approach, which has recently gained attention. However, it remains unclear which of the two options is more effective and suitable. By placing both profit-oriented and social innovators in one ecosystem, policy makers could pursue both approaches and at the same time reduce the risk that innovators, which cannot be clearly allocated to an ecosystem for profit-oriented entrepreneurs or to an ecosystem for social innovators, might become stuck between the two systems.

Second, it is unquestionable that both groups (profit-oriented entrepreneurs and social innovators) can learn from each other. It can even be argued that both groups need each other since social and environmental aspects are relevant for profit-oriented entrepreneurs while social innovators often need the technologies or entrepreneurial skills of profit-oriented entrepreneurs. It would be a valuable addition if profit-oriented entrepreneurs would consider social challenges and social innovators would make greater use of the latest technologies. Similar synergies are expected for each of the six pillars, e.g., a culture that is open to new products and services (e.g., new technologies) and at the same time considers social and environmental attributes. Third, to run supportive programs such as accelerators, a certain number of applications is needed to undertake a selection procedure and run the program in cohorts (Dempwolf et al., 2014; Pauwels et al., 2016). In regions with low population densities, extending the traditional entrepreneurial ecosystem may help to reach the critical mass necessary to run supportive programs.

Fourth, initiating an SI ecosystem from scratch is expected to require more resources (including time) than updating an existing traditional entrepreneurial ecosystem. Since many of the grand challenges must be addressed today rather than tomorrow, and due to the COVID-19 pandemic’s limited resources, updating an existing traditional entrepreneurial ecosystem appears suitable.

Despite the briefly mentioned synergies and positive effects when placing both profit-oriented entrepreneurs and social innovators in one ecosystem, tensions are expected due to the different characteristics (e.g., motivation and orientations) of the two actors. Many of these tensions can be mitigated by setting corresponding laws and regulations; however, due to difficulties in distinguishing between profit-oriented entrepreneurs and social innovators and the attendant complexity of doing so, not all tensions can be addressed and avoided. A high risk of tensions is expected in the policy field. In an ideal ecosystem, policies would be set such that they would involve both actors. In practice, however, this is not always possible. A policy favouring one type of actor can (unintentionally) hinder the other actor. For example, by introducing policies for SI education, entrepreneurial education can automatically be moved to the background. Another example involves the legal setting. If the legal setting is more suitable for one actor than for the other, tensions are likely to emerge. Access to resources is also expected to lead to tensions. The term resources must hereby be understood in a broader sense and include finances and human capital. To begin, a fair and equal distribution of financial support between profit-oriented entrepreneurs and social innovators is often tricky. The identified need to introduce suitable measures for evaluating SI impact and a governmental SI budget might be an approach to hinder possible tensions. However, obtaining a clear, unassailable intersection is never possible. Concerning human capital, social innovators and profit-oriented entrepreneurs compete for the same employees. This is particularly the case as more educated individuals strive for job positions that offer them (social) fulfilment and seek the option to leave traditional industries; therefore, this competition should not be underestimated.

Other tensions are likely to be related to support. As the number of participants in support programs offered, e.g., by hubs, is limited, profit-oriented entrepreneurs and social innovators will compete against each other. Possible solutions for mitigating tensions can relate to quotas or evaluation criteria considering social aspects. Another solution can be to offer own support programs for profit-oriented entrepreneurs and own support programs for social innovators. This can currently be observed in metropolitan regions such as New York, Vienna, and Milan, where own support programs for social innovators are offered (Center for social innovation, 2020; Domanski, 2018; Nicolopoulou et al., 2017; Sgragali and Montanari n.d.). Such a separation, however, also entails negative consequences. For example, the possible mutual learning effects between the two actors introduced above vanish. Furthermore, this approach appears unsuitable in peripheral regions where the number of actors is low.

Similar tensions are expected with regard to access to relevant infrastructure. For social innovators and profit-oriented entrepreneurs, such infrastructure involves, e.g., affordable (and often publicly funded) coworking spaces.

As the study shows, social innovators are often found in markets related to social and demographic change or environmental protection. These markets are large, and social innovators usually focus on submarkets that are different than those focused on by profit-oriented entrepreneurs. However, (financial) support for an actor in one sub-market can easily lead to unintended unfair market circumstances for the other actor. A real case example (experienced and explained by P 4) involves a voluntary driving service for elderly people in the municipality, which is supported by reimbursement of the mileage allowance from the public administration. This support led to an unintended market distortion for for-profit taxi drivers and tensions between the voluntarily drivers and for-profit taxi drivers.

## Conclusion

In this exploratory qualitative study, we shed light on the emerging needs of social innovators because they have the potential to solve today’s grand challenges and to contribute to reaching the SDGs. By analysing the 27 identified needs of social innovators, a novel SI ecosystem that partially overlaps with the traditional entrepreneurial ecosystem is introduced. Furthermore, potential synergies and tensions, including the presence of profit-oriented entrepreneurs and social innovators in one ecosystem, are briefly addressed.

The paper contributes to the SI literature by identifying the emerging needs of nonprofit-oriented innovators and social entrepreneurs and suggesting a concrete approach to fostering SI development and implementation. It furthermore introduces a novel SI ecosystem model, which, thanks to its foundation based on the traditional entrepreneurial ecosystem model, carries recognition value for many researchers.

Many regional or national policymakers, practitioners and even the European Union (Sabato et al., 2017) have recently focused on fostering SI development and implementation. Despite this increased attention, much confusion about suitable support frameworks for social innovators remains. In this regard, the study provides concrete starting points. As many cities and regions have entrepreneurial ecosystems based on Isenberg’s traditional entrepreneurial ecosystem (2010, 2011), the suggested SI ecosystem model offers policymakers and practitioners a concrete and often familiar structure for providing a supportive environment to social innovators.

As with any study, this study has a number of limitations. Due to the qualitative case study format, the findings cannot be generalized or extended to a larger population (Ochieng, 2009; Ormiston & Seymour, 2011). In addition, the region analysed in this case has the lowest population density in Austria (Mohr, 2020) and shows strong characteristics of being a peripheral region. Even though biases in selecting interview partners were avoided as much as possible, there is always a remaining bias in the sample composition (Siggelkow, 2007). Furthermore, absolute objectivity can never be achieved in the interview process due to environmental factors or the existence of a relationship between interviewer and interviewee (Qu & Dumay, 2011).

Future qualitative research is needed to verify the identified needs in other regions. More precisely, a multiple case study is suggested to obtain deeper insights into the emerging needs of social innovators and how their needs overlap and differentiate from those of profit-oriented entrepreneurs. Once extensive qualitative research has been conducted, quantitative research could contribute to the topic by identifying the importance of each need. In other words, through quantitative research, weight could be assigned to the identified needs. Additionally, the potential synergies and tensions must be investigated in future studies as the existing literature focuses exclusively on interpersonal and interorganizational synergies and tensions, e.g., the risk of mission drift (Mitzinneck & Besharov, 2019; Siebold et al., 2019; Żur, 2020). Instead, studies focusing on synergies and tensions between profit-oriented entrepreneurs and social innovators when placed in one ecosystem are still needed.

Since certain similarities between profit-oriented entrepreneurs and social innovators exist, we have anticipated the suitability of ecosystems for satisfying the emerging needs of social innovators. Regardless, gauging the success of an ecosystem approach will be the task of future studies. Future studies should focus not only on metropolitan regions but also on peripheral regions. Many peripheral regions have recently established – or are in the process of establishing – ecosystems for profit-oriented entrepreneurs. Considering the typically challenging circumstances of peripheral regions, extending the traditional entrepreneurial ecosystem based on the identified eleven unique social innovator needs holds large potential.

We suggest a multiple case study format for individuating the suitability of the introduced SI ecosystem in metropolitan and peripheral regions.

Due to the exploratory nature of this study and the missing verification of the findings through a multiple case study, this study does not intend to deliver direct policy implications. However, since in most cases the stakeholders and experts of the corresponding fields agreed with the opinions of the social innovators, a few existing approaches that have not yet received much attention but are expected to address some of the needs are briefly highlighted below.

Housing associations and public administration have begun to include “get together offices” that are run by social organizations when designing large apartment buildings. Such offices aim to connect neighbours who otherwise have mostly lived anonymously next to each other. In addition to mitigating disputes among neighbours, “get together offices” can contribute to a suitable communication and discussion culture (at least among neighbours), raise awareness about social problems and stimulate society to participate in mitigating social problems through the development of innovative ideas.

Since SIs are often developed and implemented by nonprofit-oriented innovators, it is necessary that the legal setting provide basic securities or insurance access for voluntary workers. First, federal states offer automatic accident and liability insurance for everyone who voluntarily works in the interests of the common good or addresses social concerns.

Any international (exchange) program is expected to contribute to a culture with an international horizon (e.g., student exchange, international research project). Particularly relevant are international programs focusing on SI. One existing project between four Italian and Austrian regions fosters networking and exchange among nascent social innovators and connects corresponding stakeholders (including regional politicians).

In conclusion, we would like to emphasize that the grand challenges the world is currently facing are daunting. However, recent SIs show promising results in mitigating these challenges. They give us hope that with suitable policies supporting social innovators, more SIs will emerge and the SDGs can be reached by 2030.

## Declarations

### Data availability

Available upon request and in consideration of the data privacy statement.

### Code availability

Available upon request and in consideration of the data privacy statement.
